# Influence of age, body mass index and family income on the health Quality of Life among the breast cancer patients in Bangladesh: a cross-sectional study

**DOI:** 10.3389/fonc.2026.1817273

**Published:** 2026-07-06

**Authors:** Rifat Rahman, Alokta Ishika, Tamanna Yousuf Ali, Mohammad Hayatun Nabi, Mohammad Delwer Hossain Hawladar, Koustuv Dalal

**Affiliations:** 1 KIT (Royal Tropical Institute), Amsterdam, Netherlands; 2 Department of Public Health, North South University, Dhaka, Bangladesh; 3 Khwaja Yunus Ali Medical College & Hospital (KYAMCH), Enayetpur, Chauhali, Sirajganj, Bangladesh; 4 NSU Global Health Institute (NGHI), North South University, Dhaka, Bangladesh; 5 Department of Public Health Science, School of Health Sciences, Mid Sweden University, Sundsvall, Sweden; 6 Institution for Health Sciences, University of Skövde, Skövde, Sweden

**Keywords:** Breast Cancer (BC), Quality of Life (QoL), age, income, body mass index (BMI), EORTC QLQ-C30, EORTC QLQ-BR23, Bangladesh

## Abstract

**Background:**

This study aimed to investigate the influence of BMI, age, and socioeconomic status on QoL among BC patients in Bangladesh.

**Methods:**

This cross-sectional study was conducted among 200 female breast cancer patients who had completed treatment or were undergoing follow-up at the Shanti Oncology and Palliative Care Foundation, Dhaka, Bangladesh, between May 2023 and July 2024. Participants were interviewed using validated European Organization for Research and Treatment of Cancer (EORTC) questionnaires. The QLQ-C30 for general cancer-related QoL and the BR23 module specific for breast cancer. Key independent variables included variables age, family income and BMI, assessed in relation to cancer profile, global health scores, C30-BR23 functional domains, and symptom scales. Data analysis involved descriptive statistics, assessment of normality using the Shapiro-Wilk test, and inferential analyses using logistic and linear regression models to evaluate associations between independent variables with cancer profile and QoL scale.

**Results:**

Among 200 breast cancer patients, the mean age was 49.5 years (SD 9.02), with the majority overweight (52.5%) and housewives (74%). Most had Stage II (53%) cancer, 58.5% underwent mastectomy, and 98% received chemotherapy. Global health score (GHS) was 42.44 (SD 12.46). Regression analyses identified age and family income as the most significant factors associated with QoL in this study. Older age was significantly associated with lower GHS score (β = –4.68; 95% CI: –6.68 to –2.67; p < 0.001), as well as higher insomnia (β = 6.45; 95% CI: 0.45 to 12.46; p = 0.035) and dyspnea (β = 5.64; 95% CI: 0.45 to 10.84; p = 0.033) scores. Higher family income was significantly associated with localization of BC (AOR = 1.51, 95% CI: 1.02–2.23, p = 0.038), metastasis (AOR = 2.03, 95% CI: 1.28–3.22, p = 0.003), undergoing mastectomy (AOR = 1.55, 95% CI: 1.07–2.25, p = 0.020), and receiving radiotherapy (AOR = 11.92, 95% CI: 2.34–60.63, p = 0.003). Lower family income was significantly associated with increased financial difficulties (β = –11.88; 95% CI: –16.76 to –7.00; p < 0.001), poorer future perspectives (β = –5.66; 95% CI: –10.15 to –1.16; p = 0.014), and worse breast and systemic therapy side effects (β = –5.44; 95% CI: –8.94 to –1.94; p = 0.002; β = 4.07; 95% CI: 1.23 to 6.90; p = 0.005, respectively). BMI indicated limited associations, which include increased dyspnea (β = 1.40; 95% CI: 0.15 to 2.64; p = 0.028) and decreased diarrhea scores (β = –2.11; 95% CI: –3.09 to –1.13; p < 0.001).

**Conclusion:**

The overall QoL score was poor among Bangladeshi patients. The association of BMI with C30 and BR23 was limited. In this study, age and family income were statistically significant predictors of QoL. Further studies with larger sample sizes are recommended to better explore the association between higher BMI and QoL among BR patients.

## Introduction

Breast cancer (BC) is one of the most prevalent cancers among women in more than 150 countries (1–11), with a global incidence rate of 11.6% in 2022 (1–3, 7, 8). Around 1.5 million women are diagnosed annually with BC (7, 9), and cases have been increasing in low- and middle-income countries (LMICs), particularly in Asia, over the past decade (2, 4–6, 10–18). In Bangladesh, the incidence rate of BC is 15.2%, higher than the global rate (12–15, 19, 20), with the highest prevalence observed among females aged under less than 50 years (21, 22). In 2022, approximately 72,334 new cases of BC were diagnosed in Bangladesh (19). However, the actual burden may be higher as available data may be underestimated due to the absence of a national cancer registry, limited screening programs, and incomplete clinical databases (14, 23–25). Globally, health-related quality of life (QoL) is considered a significant outcome parameter in BC patients and has been associated with treatment outcomes in cancer research (26, 27, 34, 43). Multiple factors are associated with QoL across the phases of diagnosis, care continuum, and periodic follow-up, with each phase imposing substantial physical and emotional burdens on BC survivors (22, 26–29). Studies have found that income is associated with QoL in BC patients, where higher income is linked to better QoL and lower income is associated with financial distress, limited access to cancer treatment, and poorer QoL (10, 17, 18, 26, 30–36). In Bangladesh, family income is also significantly associated with cancer treatment and QoL (16, 37). Being overweight or obese is another significant risk factor for BC (1, 4–8, 16, 22, 38, 39) and has been linked to poorer QoL (4–8, 38, 39), higher likelihood of BC (1, 4–8, 28, 38, 39) and reduced disease-free survival following treatment (4, 5, 8, 38, 39). According to the World Health Organization (WHO), about 38% of the global female population is overweight or obese (1, 4–7, 40). In Bangladesh, the prevalence of overweight or obesity among females has been reported as 45.6% (40–42). Even with advancements in cancer treatment, BC survivors are likely to experience late-emerging side effects that are associated with poorer QoL (12, 27, 39, 44–46). Despite this, the interplay of these factors and their associations with QoL remains relatively underexplored in Bangladesh (27). To the best of our knowledge, few studies in Bangladesh have examined the combined associations of BMI, age, and socioeconomic factors on QoL in BC patients using the European Organization for Research and Treatment of Cancer (EORTC) QLQ-C30 (general cancer-related QoL) and QLQ-BR23 (breast cancer–specific QoL) questionnaires. Therefore, it is important to examine the factors associated with QoL among BC patients to develop targeted interventions and improved BC patient care in Bangladesh. This study aims to examine the association of BMI, age, and socioeconomic (family income) on QoL in BC patients in Bangladesh.

## Methodology

For this cross-sectional study, patients aged above 18 years, with histopathologically confirmed BC, who were either undergoing treatment, had completed treatment, or were in post-treatment follow-up, able to respond to the questionnaire independently, registered at the chemotherapy department of the Shanti Oncology and Palliative Care Foundation (hereafter referred to as “Shanti”) in Bangladesh were recruited. Shanti, healthcare center specialized in Oncology and Cancer treatment, provides care to a diverse group of BC patients across different stages of cancer, age groups, and modes of presentation, including diagnosis of cancer and chemotherapy. Patients diagnosed with any type of cancer other than BC, those with observable deteriorated condition due to chemotherapy and those unwilling to participate were excluded from the study.

For this study, the sample size was calculated using the formula, $\text{η}={Z_{1-\frac{\alpha }{2}}}^{2}\;p(1-p)/d^{2}\;\;$, where Ƞ is the minimum sample size, Z_(1−α/2)_ = 1.96 (standard normal variate at 5% type I error, p < 0.05), p = 0.274 (27.4%) (estimated prevalence of BC from a Saudi Arabian study) (10, 47), and d = absolute error (5%). The calculated sample size was 306 participants. This study is an extended part of MPH thesis work. So, due to time constraints in data collection, the target sample size could not be achieved, and 210 participants were initially recruited. After data cleaning and exclusion of incompletely filled questionnaires, the final sample size was 200 participants. Convenience sampling was used to recruit participants. Data were collected from May 2023 to July 2024.

Data on QoL were collected using the European Organization for Research and Treatment of Cancer (EORTC) questionnaires: QLQ-C30 (general cancer-related QoL) and QLQ-BR23 (breast cancer–specific QoL). The QLQ-C30 is combination of 5 functional scales (“physical,” “cognitive,” “role,” “emotional,” and “social functioning”), 9 symptom scales (“fatigue,” “nausea/vomiting,” “pain,” “dyspnea,” “sleep disturbance,” “loss of appetite,” “constipation,” “diarrhea,” and “financial difficulties”), and a global health status (GHS) scale. Whereas the BR23 module includes 4 functional scales (“body image,” “sexual functioning,” “sexual enjoyment,” “future perspective”) and 4 symptom scales (“systemic therapy side effects,” “breast symptoms,” “arm symptoms,” and “hair loss”), all specific to BC. Both C30 and BR23 scales range from 1 to 100, with higher scores on functional scales indicating better QoL, and higher scores on symptom scales indicating more symptoms and poorer QoL. No data on comorbid conditions such as chronic obstructive pulmonary disease (COPD), diabetes mellitus, or cardiovascular diseases were collected, as these were not included as variables in this EORTC questionnaire.

**Figure 1:**
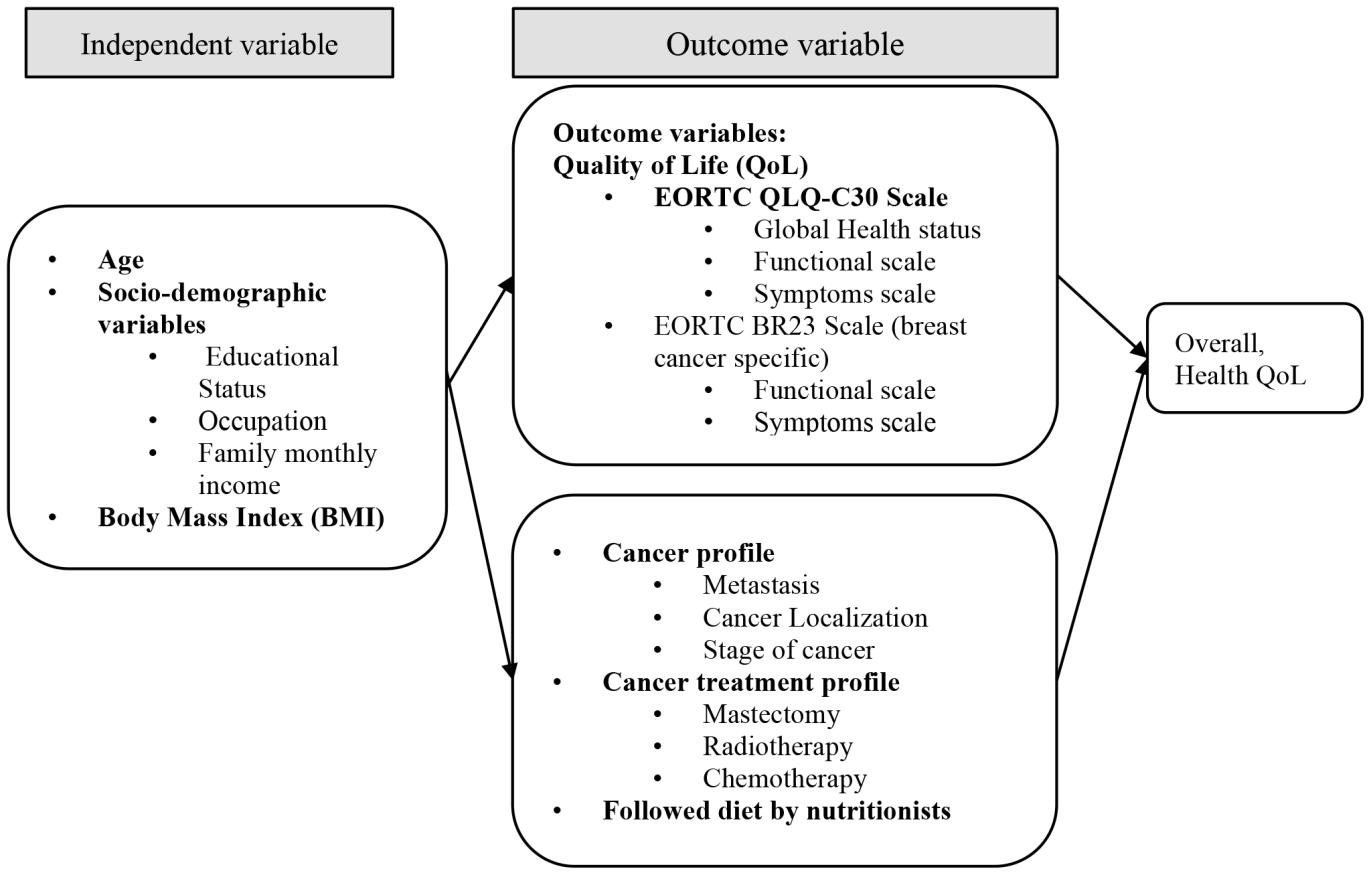
Conceptual framework of variables associated with quality of life among breast cancer patients.

Initially, the study’s purpose and objectives were explained to the participants, followed by the collection of written consent. Questionnaires were completed voluntarily or self-administered, with brief face-to-face interviews conducted as needed. Questions were explained clearly, allowing adequate time for honest responses. All data were recorded accurately, and strict confidentiality was maintained. The sociodemographic data collected included age, height, weight, educational status, employment status, and family income (**Figure 1**). For statistical analysis, age and Body Mass Index (BMI) were considered as categorical variables. BMI was calculated as weight in kilograms divided by height in meters squared (kg/m²) and categorized according to the World Health Organization (WHO) classification: underweight (<18.5), normal weight (18.5–24.9), overweight (25.0–29.9), and obese (≥30.0) for this study.

### Data analysis/statistical method

All statistical analyses were conducted using Statistical Software for Data Science (STATA 15.0) and Microsoft Excel (18.2503.1198.0). Before analysis, data were cleaned, coded, missing values addressed, and variables categorized. Descriptive analyses were utilized to summarize socio-demographic characteristics, cancer-related variables, and QoL scores from the EORTC QLQ-C30 and QLQ-BR23 questionnaires. The findings for this analysis were reported as observation numbers, means, and standard deviations (SD) for the continuous variables (EORTC QLQ-C30 and QLQ-BR23). For the categorical variables (socio-demographic characteristics, cancer-related variables), results were reported as frequencies and percentages. Data normality was assessed using the Shapiro–Wilk test. For inferential analysis, BMI, age, and socioeconomic status were considered as independent variables, while cancer profile, C30, and BR23 QoL scores were treated as outcome variables. Separate regression models were constructed for each outcome variable, and each outcome variable was analyzed independently. Binary outcome variables were analyzed using logistic regression models to estimate adjusted odds ratios (AORs) with 95% confidence intervals (CIs). Continuous outcome variables (QoL scores) were analyzed using multiple linear regression (separate for each outcome) models. Robust standard errors were used in all regression models to account for potential heteroskedasticity. A p-value < 0.05 was considered statistically significant.

## Results

Among all the BC participants (observation, N = 200), the mean age was 49.5 years (standard deviation (SD) = 9.02; range: 29–65 years), with the largest group (36%) aged 45–54 years and the smallest group (29.5%) aged over 55 years. Analyzing education, 72.5% had education beyond the primary level, while 37.5% had up to primary education. Most participants (74%) were housewives, and 45.5% of participants reported a family monthly income between 40,001 and 100,000 Bangladeshi taka (BDT) (approximately 328–822 USD, based on the September 2025 exchange rate: 1 USD = 121 BDT). The mean BMI was 25.9 (SD = 3.62; range: 17.26-38.21). More than half of participants (52.5%) were found to be overweight, 17% were obese, 37.5% had normal weight, and 3% were underweight. Nearly all the participants (99.5%) did not follow a nutritionist-guided diet. Analysis of the cancer profile revealed that more than half (53%) of the participants had Stage II BC, followed by Stage III (37.5%), and Stage IV (9%), with only one participant (0.5%) reporting Stage I. Metastases were present in 21.5% participants. As for BC treatment, 58.5% underwent mastectomy, 98% received chemotherapy, and 5.5% received radiotherapy. Detailed socio-demographic, cancer treatment, and profile analysis are presented in **Table 1**.

**Table 1:** Socio-demographic profile, BMI, and BC clinical characteristics of study participants (N = 200).

Socio-demographic characteristics	Number (N)	Frequency (%)
**Age (years)**	Mean 49.5, Range: 29-65, SD = 9.02	
<45 years	69	34.5
45 to 54 years	72	36.0
55+ years	59	29.5
**Body Mass Index (BMI) (kg/m^2^)**	Mean 25.9, Range:17.26-38.21, SD = 3.62	
Under weight	3	1.5
Normal	75	37.5
Overweight	105	52.5
Obese	17	8.5
Educational Status		
Up to Primary Education	75	37.5
Above Primary Education	125	62.5
**Occupation**		
Housewife	148	74.0
Service holder	52	26.0
**Family Monthly Income (in BDT)**	Mean: 87250, Range:10,000-5,00,000, SD = 60838.34	
Up to 20,000	9	4.5
20,001 to 40,000	41	20.5
40,001 to 100,000	91	45.5
More than 100,000	59	29.5
Cancer Localization (Yes/No)		
Yes	80	40.0
No	120	60.0
Stage of Cancer		
Stage 1	1	0.5
Stage 2	106	53.0
Stage 3	75	37.5
Stage 4	18	9.0
Followed diet by nutritionists (Yes/No)		
Yes	1	0.5
No	199	99.5
**Mastectomy Done (Yes/No)**		
Yes	117	58.5
No	83	41.5
Received Radiotherapy (Yes/No)		
Yes	11	5.5
No	189	94.5
Metastasis (Yes/No)		
Yes	43	21.5
No	157	78.5
Received Chemotherapy (Yes/No)		
Yes	196	98.0
No	4	2.0

### EORTC QLQ-C30 and BR23 score analysis

Analysis of the mean variation of EORTC QLQ-C30 and QLQ-BR23 scores (N = 200) revealed a mean global health scale (GHS) of 42.44 (SD 12.46, range: 16.6–83.3). Among the C-30 functional scales, cognitive functioning had the highest mean score (83.61, SD 23.12), and social functioning had the lowest (46.49, SD 22.93). Symptoms mean scores were highest for financial difficulties (73.67, SD 29.81) and lowest for pain (28.40, SD 25.10). In the BR-23 functioning scales, the highest mean was observed in sexual enjoyment (64.92, SD 37.18) and the lowest in future perspective (19.54, SD 26.97). On the BR-23 symptom scales, the highest mean score was for upset by hair loss (75.61, SD 25.93), and the lowest for breast symptoms (30.05, SD 20.75). Interpretation of these scales indicates that higher scores on functional scales and GHS reflect better QoL, whereas higher scores on symptom scales indicate more symptom burden and poorer QoL. Detailed mean scores with SD for QLQ-C30 and BR23 all scales are presented in **Table 2**.

**Table 2:** EORTC QLQ-C30 and BR23 mean scores and standard deviations in BC Patients (N = 200).

Scales	No of items	N	Mean	SD
QLQ-C30 questionnaire				
Global health status/QoL	2	200	42.438	12.458
Functional scales				
Physical functioning	5	200	64.666	19.709
Role functioning	2	200	70.635	22.479
Emotional functioning	4	200	53.859	34.716
Cognitive functioning	2	200	83.613	23.123
Social functioning	2	200	46.489	22.934
Symptoms scales				
Fatigue	3	200	49.959	18.293
Nausea and vomiting	2	200	43.153	21.613
Pain	2	200	28.404	25.101
Dyspnea	1	200	29.996	30.261
Insomnia	1	200	41.499	33.166
Appetite	1	200	59.613	25.867
Constipation	1	200	43.502	32.435
Diarrhea	1	200	51.666	31.660
Financial difficulties	1	200	73.667	29.813
EORTC BR23				
Function scale				
Body image	4	200	62.291	29.313
Future perspective	2	200	19.544	26.973
Sexual functioning	1	200	57.275	34.011
Sexual enjoyment	1	200	64.917	37.180
Symptom scale				
Systemic therapy side effects	7	200	49.768	17.691
Breast symptoms	4	200	30.050	20.745
Arm symptoms	3	200	42.564	28.921
Upset by hair loss	1	200	75.612	25.926

Global Health Status/QoL should not be in bold; it should be presented in regular format. It is one of the scales of the EORTC QLQ-C30 questionnaire.

The "Global Health Status / QoL" score in the EORTC QLQ-C30 represents a patient's overall quality of life.

### Logistic regression analysis of BMI, age, and family income with BC profile and treatment

Logistic regression analysis (**Table 3**) revealed that higher family monthly income was significantly associated with increased odds (1.5 times) of having localized BC (AOR = 1.51, 95% CI: 1.02–2.23, p = 0.038). BMI and age were not statistically significant with cancer localization. BC participants from higher-income families were nearly twice as likely to be associated with metastasis (AOR = 2.03, 95% CI: 1.28–3.22, p = 0.003). BMI and age were not statistically associated with metastasis (p > 0.05). BC patients from higher-income families were 1.5 times more likely to undergo a mastectomy (AOR = 1.55, 95% CI: 1.07–2.25, p = 0.020) and almost 12 times more likely to receive radiotherapy (AOR = 11.92, 95% CI: 2.34–60.63, p = 0.003). Following a diet prescribed by a nutritionist was statistically associated with age (AOR = 1.39, 95% CI: 1.00–1.93, p = 0.047), while BMI was not statistically associated (AOR = 1.08, 95% CI: 1.00–1.17, p = 0.052) and was not considered significant. Family income could not be estimated for following a diet prescribed by a nutritionist due to the limited prediction from low observation numbers. No significant associations were observed between chemotherapy and any of the predictors (p > 0.05).

**Table 3:** Logistic regression analysis of BMI, age, and family monthly income with BC profile and treatment .

Outcome	Predictor	AOR**	95% CI***	P-value
Cancer Location (Yes/No)	BMI	0.98	(0.91 – 1.06)	0.602
	Age	0.90	(0.63 – 1.28)	0.549
	Family income	1.51	(1.02 – 2.23)	0.038*
Metastasis (Yes/No)	BMI	0.94	(0.84 – 1.06)	0.316
	Age	1.28	(0.82 – 1.99)	0.276
	Family income	2.03	(1.28 – 3.22)	0.003*
Followed diet by nutritionists (Yes/No)	BMI	1.08	(1.00 – 1.17)	0.052
	Age	1.39	(1.00 – 1.93)	0.047*
Chemotherapy (Yes/No)	BMI	0.85	(0.60 – 1.20)	0.368
	Age	0.61	(0.14 – 2.59)	0.502
	Family income	2.22	(0.57 – 8.58)	0.248
Mastectomy (Yes/No)	BMI	0.96	(0.89 – 1.04)	0.337
	Age	1.13	(0.79 – 1.63)	0.506
	Family income	1.55	(1.07 – 2.25)	0.020*
Radiotherapy (Yes/No)	BMI	0.80	(0.63 – 1.01)	0.061
	Age	1.44	(0.63 – 3.30)	0.391
	Family income	11.92	(2.34 – 60.63)	0.003*

*p value < 0.05; **AOR, Adjusted Odds Ratio; ***CI, Confidence interval.

### Linear regression analysis of BMI, age, and family income on cancer profile and QoL (EORTC QLQ-C30/BR23)

Multiple linear regression analyses (**Table 4**) revealed that higher family income was associated with cancer stage (β = 0.243, 95% CI: 0.127 to 0.359, p < 0.001), indicating that BC patients from higher-income families were more likely to present with advanced stages of BC. Age and BMI were not statistically associated with cancer stage (p > 0.05).

**Table 4:** Multiple Linear regression (separate) of BMI, age, and family income with cancer profile and EORTC QLQ-C30 and QLQ-BR23 scores.

Scale			BMI			Age			Family Monthly Income		
		Outcome	β (Coef.)**	95% CI***	p-value	β (Coef.) **	95% CI***	p-value	β (Coef.)**	95% CI***	p-value
	Cancer Profile	Cancer Stage	-0.001	(-0.025, 0.023)	0.916	0.115	(-0.003, 0.233)	0.056	0.243	(0.127, 0.359)	<0.001*
EORTC QLQ-C30 (General Questionnaire for Cancer patients)	Global Health Score	QoL/Global Health Score	0.330	(-0.180, 0.841)	0.203	-4.676	(-6.683, -2.669)	<0.001*	-2.072	(-4.087, -0.058)	0.044*
	Functional scale	Physical Functioning	-0.141	(-0.903, 0.620)	0.715	-9.512	(-12.909, -6.114)	<0.001*	-1.337	(-4.206, 1.533)	0.359
		Role Functioning	0.355	(-0.614, 1.325)	0.471	-4.877	(-8.844, -0.910)	0.016*	-0.579	(-4.476, 3.317)	0.770
		Emotional Functioning	0.007	(-1.144, 1.158)	0.991	-5.051	(-11.313, 1.210)	0.113	3.946	(-1.841, 9.733)	0.180
		Cognitive Functioning	-0.351	(-1.329, 0.628)	0.481	-4.064	(-7.877, -0.250)	0.037*	5.505	(1.238, 9.773)	0.012*
		Social Functioning	0.172	(0-.814, 1.158)	0.731	-3.166	(-7.367, 1.038)	0.139	-2.953	(-6.869, 0.962)	0.138
	Symptom scale	Fatigue	0.018	(-0.735, 0.772)	0.962	4.351	(1.122, 7.580)	0.009*	1.877	(-1.179, 4.933)	0.227
		Nausea and vomiting	-0.023	(0-.915, 0.868)	0.959	2.004	(-1.808, 5.816)	0.301	-2.806	(-6.766, 1.154)	0.164
		Pain	- 0.102	(-1.220, 1.017)	0.858	2.780	(-1.478, 7.038)	0.199	-6.681	(-10.832, -2.530)	0.002*
		Dyspnea	1.398	(0.153, 2.643)	0.028*	5.644	(0.447, 10.840)	0.033*	-9.948	(-14.468, -5.428)	<0.001*
		Insomnia	-0.128	(-1.497, 1.242)	0.854	6.454	(0.448,12.461)	0.035*	-3.527	(-8.741,1.687)	0.184
		Appetite loss	-0.079	(-1.146,0.987)	0.884	3.384	(-1.262,8.030)	0.152	3.328	(-1.070,7.726)	0.137
		Constipation	0.437	(-1.035,1.910)	0.559	5.735	(0.046,11.423)	0.048*	1.918	(-3.680,7.517)	0.500
		Diarrhea	-2.110	(-3.089, -1.132)	<0.001*	0.011	(-5.210, 5.232)	0.997	15.750	(11.168, 20.331)	<0.001*
		Financial Difficulties	-0.527	(-1.654, 0.601)	0.358	2.397	(-2.539, 7.333)	0.339	-11.879	(-16.758, -7.000)	<0.001*
EORTC QLQ-BR23 (Breast Cancer Specific Questionnaire)	Functional scale	Body image	-0.047	(-1.183,1.090)	0.935	3.259	(-1.780,8.298)	0.204	0.066	(-5.267 – 5.399)	0.980
		Future perspective	-0.392	(-1.574,0.790)	0.514	-2.072	(-6.495,2.350)	0.357	-5.657	(-10.151, -1.163)	0.014*
		Sexual functioning	0.688	(-0.647,2.024)	0.311	-5.258	(-11.977,1.462)	0.124	-4.758	(-10.305,0.788)	0.092
		Sexual enjoyment	1.095	(-0.269,2.460)	0.115	-14.069	-21.046, -7.092	<0.001*	-3.467	(-9.426,2.492)	0.253
	Symptom scale	Systemic therapy side effects	-0.043	(-0.755,0.670)	0.906	1.873	(-1.187,4.932)	0.229	4.068	(1.232,6.904)	0.005*
		Breast symptoms	-0.038	(-0.938, 0.862)	0.934	-0.017	(-3.659, 3.624)	0.993	-5.438	(-8.936, -1.939)	0.002
		Arm Symptoms	-1.081	(-2.180, 0.017)	0.054	4.767	(-0.333, 9.867)	0.067	6.657	(1.870, 11.443)	0.007
		Upset by hair loss	-0.458	(-1.592,0.675)	0.426	-2.066	(-6.495,2.362)	0.359	7.782	(3.348,12.217)	0.001*

*p < 0.05 (statistically significant); ** β (Coef.) = Regression coefficient; *** CI = Confidence interval.

For the EORTC QLQ-C30 global health scale (GHS), age was inversely associated with GHS (β = -4.676, 95% CI: -6.683 to -2.669, p < 0.001), indicating that with increasing age, BC patients were likely to have a lower GHS score (lower QoL). Family income was also inversely associated (β = -2.072, 95% CI: -4.087 to -0.058, p = 0.044) with GHS, indicating that BC patients from higher-income families were likely to have a lower perceived health. BMI was not significantly associated with GHS. Analysis of the EORTC QLQ-C30 functional scales indicated an inverse association between age and physical functioning (PF) (β = -9.512, 95% CI: -12.909 to -6.114, p < 0.001), indicating that PF is likely to decline with increasing age. Age was also inversely associated with Role functioning (RF) (β = -4.877, 95% CI: -8.844 to -0.910, p = 0.016), indicating that with advancing age, RF was likely to decrease, which is associated with poorer QoL. BMI and family income were not significantly associated with PF or RF. Age was inversely associated with Cognitive functioning (CF) (β = -4.064, 95% CI: -7.877 to -0.250, p = 0.037), indicating deterioration in CF likely with increasing age. In contrast, family income was associated with CF (β = 5.505, 95% CI: 1.238 to 9.773, p = 0.012), implying that BC patients from higher-income families were likely to have better CF, associated with higher QoL. No predictors were significantly associated with social functioning (SF) or Emotional functioning (EF).

As for the symptoms scale of QLQ-C30, fatigue was associated with age (β = 4.351, 95% CI: 1.122 to 7.580, p = 0.009), suggesting that older BC patients were likely to have higher fatigue levels. Family income was inversely associated with pain (β = -6.681, 95% CI: -10.832 to -2.530, p = 0.002), indicating that patients from higher-income families were less likely to experience pain. BMI, age, and family income were significantly associated with dyspnea. BMI was associated with dyspnea (β = 1.398, 95% CI: 0.153 to 2.643, p = 0.028), suggesting that BC patients with higher BMI are more likely to experience breathing difficulties. Age was also associated with dyspnea (β = 5.644; 95% CI: 0.447, 10.840; p = 0.033), implying that respiratory symptoms were likely to increase with advancing age. Family income was inversely associated with dyspnea (β = -9.948, 95% CI: -14.468 to -5.428, p < 0.001), suggesting that patients from higher-income households were less likely to experience respiratory symptoms. Age was associated with insomnia (β = 6.454; 95% CI: 0.448, 12.461; p = 0.035), and constipation (β = 5.735; 95% CI: 0.046, 11.423; p = 0.048), indicating that with increasing age, BC patients were more likely to experience sleep disturbances and constipation.

BMI was inversely associated with diarrhea (β = -2.110; 95% CI: -3.089 to -1.132; p < 0.001), indicating that patients with higher BMIs were less likely to report diarrheal symptoms. In contrast, family income was statistically associated with diarrhea (β = 15.750; 95% CI: 11.168-20.331; p < 0.001), indicating a higher likelihood of diarrhea among patients from higher-income families. Higher family income was inversely associated with financial difficulties (β = -11.879, 95% CI: -16.758 to -7.000, p < 0.001), indicating that BC patients from higher-income families were less likely to experience financial strain related to their BR-related symptoms. BMI was not significantly associated with the QLQ-C30 symptom scale variables, which included fatigue, nausea and vomiting, pain, insomnia, appetite loss, constipation, or financial difficulties. Likewise, age showed no significant association with nausea and vomiting, pain, appetite loss, diarrhea, or financial difficulties.

Multiple linear regression analysis (**Table 4**) also revealed that, among the QLQ-BR23 functional scales, family monthly income was inversely associated with future perspective (β = −5.657, 95% CI: −10.151 to −1.163, p = 0.014), indicating that BC patients from higher-income families were more likely to have a less optimistic outlook on their future. Age was inversely associated with sexual enjoyment (β = −14.069, 95% CI: −21.046 to −7.092, p < 0.001), indicating that older BC patients were more likely to experience lower sexual enjoyment. Other functional scale factors, including body image and sexual functioning, showed no statistically significant associations with BMI, age, or family income.

For symptom scales of QLQ- BR23, family monthly income was statistically associated with systemic therapy side effects (β = 4.068, 95% CI: 1.232 to 6.904, p = 0.005), indicating that BC patients from higher income families were more likely to experience BC treatment-related side effects. Similarly, family monthly income was associated with arm symptoms (β = 6.657, 95% CI: 1.870 to 11.443, p = 0.007), implying that BC patients from high-income families were more likely to experience more arm symptoms. Family monthly income was also associated with upset due to hair loss (β = 7.782, 95% CI: 3.348 to 12.217, p = 0.001), suggesting that BC patients from high-income families are more likely to be distressed by hair loss. In contrast, family monthly income was inversely associated with breast symptoms (β = -5.438, 95% CI: -8.936 to -1.939, p = 0.002), indicating that BC patients coming from high-income families were less likely to experience breast symptoms. BMI showed no significant associations across all BR23 function and symptoms scales.

## Discussions

According to the EORTC scoring guidelines, the scales range from 1 to 100. Higher scores on the functional scales of the QLQ-C30, BR23, and the global health status (GHS) scale indicate better QoL, whereas higher scores on the symptom scales of the QLQ-C30 and BR23 indicate more symptoms and poorer QoL (27). In the present study, the mean GHS score was 42.44, which is lower than the broader range reported in other global studies (56.32–72.48) (10, 17, 28) and lower than that reported in a Bangladeshi study, where the mean GHS score was 49.10 for females (27, 28).

Analysis of the QLQ-C30 functional scale revealed that cognitive functioning scored highest (83.61) and social functioning lowest (46.49). These findings suggest that while cognitive abilities remain relatively unaffected, patients with BC are likely to be withdrawn from social interactions. This could be attributable to poor social support and may also be linked to stigma or social perceptions of BC in Bangladesh (32). Global studies have signified the importance of social support from family and society as a key factor in improving QoL for BC patients (10, 26–29). On the QLQ-C30 symptom scale, financial difficulties scored the highest (73.67) and pain the lowest (28.40). These findings suggest that financial burden may be a major stressor for BC patients in Bangladesh, where health financing protection is limited and out-of-pocket expenditures are common. Financial difficulties were also identified as a significant factor associated with QoL in another study conducted in Bangladesh (27).

On the QLQ-BR23 functioning scale, sexual enjoyment (64.92) and body image (62.29) scored the highest, indicating a likely better sexual life and body image perception within this cohort. Conversely, the lowest score was observed for future perspective (19.54), suggesting that side effects of BC treatment and inherent uncertainty of living with BC are likely to impact survivors’ outlook on their future, associated with poorer QoL. On the QLQ-BR23 symptom scale, upset by hair loss scored the highest (75.61), and breast symptoms scored the lowest (30.05). Hair loss is one of the most visible and distressing common treatment side effects of BC, often associated with reduced confidence in appearance-conscious cultural contexts associated with poorer QoL (28). By comparison, the relatively lower score for breast symptoms suggests that, although physical discomfort is present, it may likely be perceived as less socially visible and thus may affect daily functioning differently than highly noticeable changes such as hair loss.

### Age

Logistic regression analysis highlighted the association between age and cancer profile. The QLQ-C30 and QLQ-BR23 scales revealed that age was statistically associated with diet, followed by nutritionists, suggesting that older patients may be more likely to follow dietary guidance, potentially due to better comorbidity management. Multiple linear regression analyses revealed age as a statistically significant factor for this study. Age was inversely associated with GHS, physical, role, and cognitive functioning on the QLQ-C30 functional scale. These findings indicate that with increasing age, BC patients were likely to have reduced physical activity, impaired role performance, and cognitive challenges, such as memory or concentration difficulties, which are associated with poorer QoL, consistent with other studies (22, 28). On the QLQ-C30 symptom scale, age was associated with fatigue, dyspnea, insomnia, and constipation, indicating that older BC patients were more likely to experience these debilitating symptoms more frequently as a result of both the disease and treatment, which may adversely affect their day-to-day functioning and wellbeing and is associated with poorer QoL, in line with other study findings (28). These results differ from a study conducted in Bangladesh, where appetite loss, nausea and vomiting, and pain were reported as the more severe symptoms associated with poorer QoL (27). For the QLQ-BR23 functioning scale, age was inversely associated with sexual enjoyment, indicating that older BC patients were likely to have limited sexual enjoyment, potentially attributed to both age-related physiological changes and the cumulative emotional toll of BC. This finding also aligns with the previous studies reviewed (10, 28), where it was highlighted that intimacy can be affected by the disease itself or its treatment, body image can be disturbed, and sexual dysfunction can be aggravated by conditions that arise from breast cancer or its treatments (28, 29).

### Family monthly income

Logistic regression analysis revealed that higher family income was statistically associated with a greater likelihood of cancer localization, metastasis, and receiving advanced treatments, such as mastectomy and radiotherapy. This implies that patients with higher incomes are more likely to have greater opportunities for early detection due to better access to diagnostic services and greater affordability, which is associated with higher QoL. In the linear regression analysis on the QLQ-C30 scale, family income was inversely associated with GHS, suggesting that patients from higher-income families are more likely to have poorer QoL. Family income was associated with cognitive functioning. On the QLQ-C30 symptom scale, higher family income was associated with less pain, less dyspnea, and fewer financial difficulties; however, it was also associated with a higher likelihood of experiencing diarrhea. These findings can be inferred to reflect that BC patients from higher-income families are more likely to have better access to pain and symptom management, receive advanced treatments and, improved cognitive functioning (17, 34). This current study was also consistent with other studies, which have found that higher income is associated with several features of improved patient care, including faster treatment, access to better rehabilitation, and reduced concern about the financial consequences of treatment (17, 34, 35, 48). However, exposure to more intensive BC treatment and its side effects may contribute to gastrointestinal symptoms, while elevated expectations and psychosocial pressures associated with maintaining socioeconomic status may likely be associated with poorer QoL (34, 48). For the QLQ-BR23, higher family income was inversely associated with future perspective and breast symptoms, and was statistically associated with systemic therapy side effects, arm symptoms, and upset due to hair loss. This finding suggests that BC patients from higher-income families were more likely to have better access and affordability to BC treatments and management; therefore, this could be associated with more systemic therapy side effects due to undergoing more BC treatment, and potentially leading to more systemic therapy side effects. Hair loss is a common side effect of breast cancer treatment, and arm symptoms may result from surgery. Studies have reported hair loss and the side effects of systemic therapy as the most distressing symptoms (10). In contrast, high-income group BC patients are likely to experience more breast-related physical symptoms, which could be attributable to access to better surgical or reconstructive care, and is likely to be associated with poor future perspectives and poorer QoL (10).

### BMI

Logistic regression found no association between BMI and cancer profile or treatment. Linear regression analysis revealed limited associations with QoL (dyspnea and diarrhea) on the QLQ-C30 scale. BMI showed no significant associations with BR23 functional or symptom scales.

These results highlight that age and family income are significantly associated with cancer profile, cancer treatment, and patient-reported QoL among breast cancer survivors. Interventions to improve QoL should prioritize symptom management for older patients and support for lower-income patients who may face financial and treatment-related challenges. Future studies should investigate the mechanisms underlying these associations and explore targeted strategies to improve equitable care delivery.

## Limitations

While this study provides novel insights into the association of age, socioeconomic status, and BMI with QoL among BC patients in Bangladesh, several limitations should be acknowledged. First, data were collected from a single private cancer care institution, which, although it draws patients from across the country, may limit the generalizability of the findings to the wider population. Second, this cross-sectional methodological design significantly relies on self-reported socio-reproductive information, which may be affected by recall bias or cultural stigma, particularly for sensitive topics such as body image, sexual enjoyment, and sexual functioning, potentially leading to misclassification or underreporting. Additionally, for the socioeconomic indicator, this study only considered family monthly income/household income in inferential analysis, while education and occupation were analyzed descriptively. Taking socioeconomic status into account would have enabled a more comprehensive explanation of the complex influence. Although the targeted sample size was not achieved, the final sample size remains sufficient to support the primary findings of the study. Despite these limitations, the findings provide valuable insights into how age, family income, and BMI are associated with QoL and may inform future research in low-resource settings such as Bangladesh. Future multidisciplinary studies with larger and more diverse samples are recommended to assess the consistency of these findings across different healthcare settings.

## Conclusions

This study highlighted that the overall QoL score was poor among Bangladeshi breast cancer patients. Increasing age was associated with advanced stages of cancer and poorer QoL in both the QLQ-C30 and QLQ-BR23 scales. Family income was also significantly associated with both the C30 and BR23 scales, with higher-income BC patients more likely to have localized disease, better access to pain and symptom management, and to receive advanced treatments, and to report improved cognitive functioning. However, higher-income patients were also more likely to experience breast-related physical symptoms. The association between BMI and both C30 and BR23 was limited, and, in this study, BMI was not a major determinant of QoL. However, existing evidence suggests that higher BMI is associated with poorer QoL as well as increased symptom burden among BR patients. Therefore, further studies with larger sample sizes are recommended to better explore the association between higher BMI and QoL among BR patients.

## Data Availability

The raw data supporting the conclusions of this article will be made available by the authors, without undue reservation.
